# An Increase in CD3+CD4+CD25+ Regulatory T Cells after Administration of Umbilical Cord-Derived Mesenchymal Stem Cells during Sepsis

**DOI:** 10.1371/journal.pone.0110338

**Published:** 2014-10-22

**Authors:** Yu-Hua Chao, Han-Ping Wu, Kang-Hsi Wu, Yi-Giien Tsai, Ching-Tien Peng, Kuan-Chia Lin, Wan-Ru Chao, Maw-Sheng Lee, Yun-Ching Fu

**Affiliations:** 1 Institute of Medicine, Chung Shan Medical University, Taichung, Taiwan; 2 Department of Pediatrics, Chung Shan Medical University Hospital, Taichung, Taiwan; 3 School of Medicine, Chung Shan Medical University, Taichung, Taiwan; 4 Department of Pediatrics, Taichung Tzuchi Hospital, the Buddhist Medical Foundation, Taichung, Taiwan; 5 Department of Medicine, Tzu Chi University, Hualien, Taiwan; 6 School of Chinese Medicine, China Medical University, Taichung, Taiwan; 7 Department of Hemato-oncology, Children's Hospital, China Medical University Hospital, China Medical University, Taichung, Taiwan; 8 Departments of Pediatrics, Changhua Christian Hospital, Changhua, Taiwan; 9 School of Medicine, Kaohsiung Medical University, Kaohsiung, Taiwan; 10 Department of Biotechnology and Bioinformatics, Asia University, Taichung, Taiwan; 11 School of Nursing, National Taipei University of Nursing and Health Sciences, Taipei, Taiwan; 12 Life-Course Epidemiology and Human Development Research Group, National Taipei University of Nursing and Health Sciences, Taipei, Taiwan; 13 Department of Pathology, Chung Shan Medical University Hospital, Taichung, Taiwan; 14 Department of Obstetrics and Gynecology, Chung Shan Medical University Hospital, Taichung, Taiwan; 15 Institute of Clinical Medicine, National Yang-Ming University, Taipei, Taiwan; 16 Department of Pediatrics, Taichung Veterans General Hospital, Taichung, Taiwan; Centre de Recherche Public de la Santé (CRP-Santé), Luxembourg

## Abstract

Sepsis remains an important cause of death worldwide, and vigorous immune responses during sepsis could be beneficial for bacterial clearance but at the price of collateral damage to self tissues. Mesenchymal stem cells (MSCs) have been found to modulate the immune system and attenuate sepsis. In the present study, MSCs derived from bone marrow and umbilical cord were used and compared. With a cecal ligation and puncture (CLP) model, the mechanisms of MSC-mediated immunoregulation during sepsis were studied by determining the changes of circulating inflammation-associated cytokine profiles and peripheral blood mononuclear cells 18 hours after CLP-induced sepsis. In vitro, bone marrow-derived MSCs (BMMSCs) and umbilical cord-derived MSCs (UCMSCs) showed a similar morphology and surface marker expression. UCMSCs had stronger potential for osteogenesis but lower for adipogenesis than BMMSCs. Compared with rats receiving PBS only after CLP, the percentage of circulating CD3+CD4+CD25+ regulatory T (Treg) cells and the ratio of Treg cells/T cells were elevated significantly in rats receiving MSCs. Further experiment regarding Treg cell function demonstrated that the immunosuppressive capacity of Treg cells from rats with CLP-induced sepsis was decreased, but could be restored by administration of MSCs. Compared with rats receiving PBS only after CLP, serum levels of interleukin-6 and tumor necrosis factor-α were significantly lower in rats receiving MSCs after CLP. There were no differences between BMMSCs and UCMSCs. In summary, this work provides the first in vivo evidence that administering BMMSCs or UCMSCs to rats with CLP-induced sepsis could increase circulating CD3+CD4+CD25+ Treg cells and Treg cells/T cells ratio, enhance Treg cell suppressive function, and decrease serum levels of interleukin-6 and tumor necrosis factor-α, suggesting the immunomodulatory association of Treg cells and MSCs during sepsis.

## Introduction

Even with standard therapeutic approaches, sepsis remains an important cause of mortality worldwide [1]. Under such conditions, vigorous immune responses could be beneficial for bacterial clearance. However, the hyperactive and out-of-balance network of cytokines may lead to tissue damage, multiple organ dysfunction and even death. Therefore, it is important to examine innovative and efficacious strategies to bring the immune responses back into balance to ultimately improve outcomes.

Mesenchymal stem cells (MSCs) have been a promising platform for cell-based therapy over the last decade. Apart from their capacity to differentiate into a variety of cell lineages and their clinical interest in tissue repair [2], MSCs have emerged as potent immune regulators [3]–[8]. Being receptive to excessive inflammation, MSCs would orchestrate the pathogen clearance through promotion of immune cell survival and function followed by suppression of the immune responses in the resolution of inflammation. Several studies demonstrated the beneficial effects of MSCs in septic animals [9]–[12], but the mechanisms of MSC-mediated regulation during sepsis are not fully elucidated.

In the present study, the immunomodulatory properties of MSCs were investigated using a well-established cecal ligation and puncture (CLP) murine model of polymicrobial sepsis. The mechanisms were studied by determining the changes of circulating inflammation-associated cytokine profiles and peripheral blood mononuclear cells after MSC administration during sepsis. Due to the limited data available regarding umbilical cord-derived MSCs (UCMSCs) for sepsis, MSCs derived from bone marrow and umbilical cord were used and compared.

## Materials and Methods

### Isolation of MSCs from bone marrow

The study was approved by the institutional review board of the Chung Shan Medical University Hospital (CSMUH No: CS13157). Bone marrow cells were obtained from iliac crest aspirates of healthy donors with written informed consents. Bone marrow-derived MSCs (BMMSCs) were isolated and cultured as our previous reports [13], [14]. In brief, mononuclear cells were isolated by Ficoll-Paque density gradient centrifugation (1.077 g/ml; Amersham Biosciences, Uppsala, Sweden), and then seeded in low-glucose DMEM (Gibco, Gaithersburg, MD) supplemented with 10% fetal bovine serum (FBS; Gibco, Gaithersburg, MD) and 1% antibiotic-antimycotic (Gibco, Gaithersburg, MD). Cells were incubated at 37°C with 5% CO_2_ in a humidified atmosphere. After 48 hours, non-adherent cells were washed out, and culture medium was changed twice per week thereafter.

### Isolation of MSCs from umbilical cords

UCMSCs were collected and isolated as our previous reports [14]–[16]. Briefly, umbilical cord was obtained from full-term infants immediately after birth with written informed consents from the parents. The cord blood vessels were carefully removed to retain Wharton's jelly. Wharton's jelly was digested in 1 mg/ml collagenase (Sigma, St. Louis, MO), and then placed in α-MEM (Gibco, Carlsbad, CA) supplemented with FBS and antibiotic-antimycotic. After culture for 48 hours, medium with suspension of non-adherent cells was discarded and medium was replaced twice a week thereafter.

### Identification of MSCs

When reaching 80–90% confluence, cultured cells were detached with trypsin-EDTA (Gibco, Carlsbad, CA) and replated at a density of 6×10^3^ cells/cm^2^ for subculture. MSCs, either BMMSCs or UCMSCs, of passage 5 were used for further studies.

To evaluate the expression of surface markers, cultured MSCs were detached, washed, and resuspended in phosphate-buffered saline (PBS; Gibco, Gaithersburg, MD). After fixing and blocking, the cells were immunolabeled with FITC or PE conjugated mouse antihuman antibodies specific to CD34, CD45, CD14, CD29, CD44, CD73, CD90, CD105, HLA-A, HLA-B, HLA-C or HLA-DR. The nonspecific mouse IgG served as isotype control. All reagents were purchased from BD Biosciences. Data were analyzed by flow cytometry (FACSCalibur; BD Biosciences, San Jose, CA) with CellQuest software.

To evaluate differentiation potential, cultured MSCs were detached from culture dishes and replated in 60-mm dishes. For induction of osteogenesis, MSCs were grown in DMEM with 10% FBS, 10 mM β-glycerophosphate (Sigma, St Louis, MO), 0.1 µM dexamethasone (Sigma, St Louis, MO), and 0.2 mM ascorbic acid (Sigma, St Louis, MO). After 2 weeks, osteogenic differentiation was demonstrated by mineralized deposits stainable with von Kossa stain (Cedarlane, Ontario, Canada). To promote adipogenesis, MSCs were incubated in DMEM with 10% FBS, 1 µM dexamethasone, 0.5 mM 3-isobutyl-1-methylxanthine (Sigma, St Louis, MO), 0.1 mM indomethacin (Sigma, St Louis, MO), and 10 µg/ml insulin (Novo Nordisk A/S, Bagsværd, Denmark). After 2 weeks, adipogenic differentiation was demonstrated by intracellular accumulation of lipid droplets stainable with oil red O (Sigma, St Louis, MO).

### CLP Model of polymicrobial sepsis in rats

The experimental protocol was approved by the Institutional Animal Care and Use Committee of the Chung Shan Medical University Experimental Animal Center (No: 1430). Male immune competent Wistar rats weighing 250 to 300 g were provided by the National Science Council. CLP, a well-established murine model of polymicrobial sepsis, leads to a focal inflammation and subsequently becomes systemic rapidly as a consequence of continuous dissemination of endogenous intestinal bacteria. This model closely resembles the septic process in humans [17], [18], and thus was employed in our study. Briefly, rats were anesthetized by intramuscular injection of 75 mg/kg ketamine and 5 mg/kg xylazine. After laparotomy, the distal one half of the cecum was ligated with a 4–0 silk tie. A single through-and-through perforation was made in the ligated segment with a 18-gauge needle and a 1 mm column of fecal material was extruded through the puncture site. Then the cecum was replaced into abdomen and the abdominal incision was closed in two layers with 3–0 silk sutures. Sham-operated rats underwent the same procedure, including opening the peritoneum and exposing the bowel, but without ligation and needle perforation of the cecum.

### Administration of MSCs after CLP

To assess the effects of MSC administration after CLP-induced polymicrobial sepsis, rats of BMMSC and UCMSC groups received five millions BMMSCs and UCMSCs in 0.3 ml sterile PBS via the tail vein 4 hours after CLP, respectively. In vitro cultured BMMSCs and UCMSCs of passage 5 were used in this study. In PBS group and sham control group, sterile PBS in a volume of 0.3 ml with no cells was administered at the same time point. Depending on the experiment, rats were either euthanized at 18 hours after surgery to harvest blood and organs, or were observed every 6 hours for 14 days to determine survival.

### Determination of serum cytokine levels

After sacrificed at 18 hours after CLP or sham operation, blood was collected and then serum was separated by centrifugation at 10,000 g for 10 min at 4°C, aliquoted, and stored at −80°C until assayed. For determination of circulating cytokine levels, the concentrations of granulocyte-macrophage colony-stimulating factor (GM-CSF), monocyte chemotactic protein (MCP)-1, interleukin (IL)-1, IL-6, tumor necrosis factor-α (TNF-α), and IL-10 were measured using bead-based multiplex immunoassays with flow cytometry (eBioscience FlowCytomix; Bender MedSystems, Vienna, Austria), according to the manufacturer's instructions.

### Analysis of peripheral blood mononuclear cells

After sacrificed, one part of blood collected in a EDTA-containing tube was used for analysis of peripheral blood mononuclear cells by their cell surface markers. Flow cytometry was performed on FACSCalibur with CellQuest software following the manufacturer's instructions. FITC, PE, or APC conjugated monoclonal antibodies specific to rat CD3, CD4, CD8a, CD11b, CD11b/c, CD25, Gran, CD45RA, or CD161a were employed with appropriate isotype matched controls. All reagents were purchased from BD Biosciences.

### Assessment of regulatory T (Treg) cell function

Spleens were harvested after sacrificed at 18 hours after CLP or sham operation. They were cut into pieces, milled with tissue grinder, and filtered. Red blood cells were removed, and splenocytes were isolated by Ficoll-Paque gradient centrifugation (GE Healthcare, Uppsala, Sweden). CD4+CD25+ and CD4+CD25- cells were isolated using CD4+CD25+ Treg cell isolation kits (Miltenyi Biotec, Bergisch Gladbach, Germany). The purity of the CD4+CD25+ T cell population analyzed by flow cytometry was greater than 95%. To evaluate the suppressive capacity of Treg cells, carboxyfluorescein succinimidyl ester (CFSE; Invitrogen, Carlsbad, CA)-labeled CD4+CD25- cells were stimulated by a IL-2 (1000 U/ml)/CD3 (3.75 µg/ml)/CD28 (3 µg/ml) mixture (BD Pharmigen, San Diego, CA) at the density of 1×10^6^ cells/well in 24-well plates for 3 days. Then isolated CD4+CD25+ Treg cells (1×10^5^ cells/well) were added into the well and cocultured for 3 days. Proliferation of CFSE-labeled CD4+CD25- cells were rated using flow cytometry (FC500, Beckman Coulter, Fullerton, CA), as previous described [19].

### Detection of the transferred human MSCs in recipient rats

For detection of the transferred human MSCs in the peripheral blood of the recipient rat, one part of blood was collected in a tube containing EDTA immediately after sacrificed. Red blood cells were lysed by RBC Lysis Solution (Qiagen, Foster, CA), and cells were collected as a pellet after centrifugation. The cells were washed and resuspended in PBS, and then immunolabeled with purified mouse antihuman APC-conjugated CD44 or PE-conjugated CD105 monoclonal antibody (BD Pharmigen, San Diego, CA). Data were analyzed by flow cytometry following the manufacturer's instruction.

For detection of the transferred MSCs in the lung of the recipient rat, the lung tissue was fixed in 10% neutral buffered formalin and then embedded in paraffin. Thin sections of 5 µm thickness were obtained for immunohistochemical staining specific to human CD44 and CD105 (GeneTex, Irvine, CA), according to the manufacturer's protocol.

### Statistical analysis

Data analysis was performed using SPSS 16.0 for Windows. Survival was analyzed with Kaplan-Meier survival curves. For continuous variables, Kruskal-Wallis test was used to compare groups and Games-Howell test was as post-hoc test. Statistical value of *p*<0.05 was considered to be significant.

## Results

### Characteristics of MSCs

**Figure 1:**
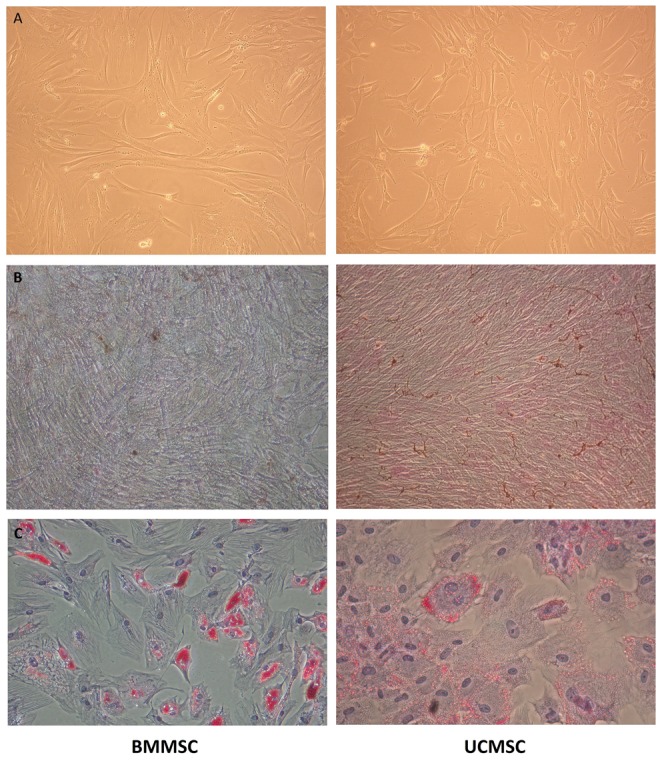
Comparison of BMMSCs and UCMSCs. (**A**) In vitro culture, BMMSCs and UCMSCs showed a similar spindle-shaped morphology (100×). (**B**) UCMSCs had stronger potential for osteogenesis than BMMSCs after 2-week induction (von Kossa staining, 100×). (**C**) UCMSCs had lower potential for adipogenesis than BMMSCs after 2-week adipogenic induction (Oil red O staining, 200×).

In vitro culture, BMMSCs and UCMSCs showed a similar spindle-shaped morphology (Figure 1A). Both revealed a consistent immunophenotypic profile which was negative for CD34, CD45, CD14, and HLA-DR, and positive for CD29, CD44, CD73, CD90, CD105, HLA-A, HLA-B, and HLA-C. There was no significant difference in the expression level of any single surface marker between BMMSCs and UCMSCs. Under respective induction conditions, both BMMSCs and UCMSCs can achieve osteogenic and adipogenic differentiation. It is interesting to note that UCMSCs had significantly stronger potential for osteogenesis but lower for adipogenesis, as shown by more intense von Kossa stain (Figure 1B) and less intense Oil red O stain (Figure 1C).

### Survival study

**Figure 2:**
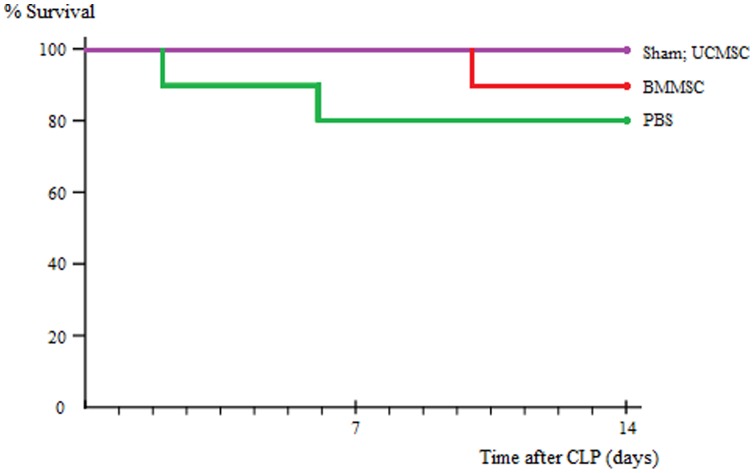
The beneficial effects from MSC administration on survival in rats with CLP-induced sepsis. Survival was evaluated for 14 days. Although no statistical significance, it appeared that rats receiving UCMSCs or BMMSCs after CLP had lower mortality rates than rats receiving PBS only after CLP. n = 10 rats/group.

Figure 2 shows the beneficial effects from MSC administration on survival after CLP-induced sepsis in rats. In the absence of antibiotics therapy, the mortality rates in rats receiving UCMSCs or BMMSCs after CLP were reduced compared with rats receiving PBS only after CLP although no statistical significance.

### Changes of cytokine profiles in rats with sepsis after MSC administration

**Figure 3:**
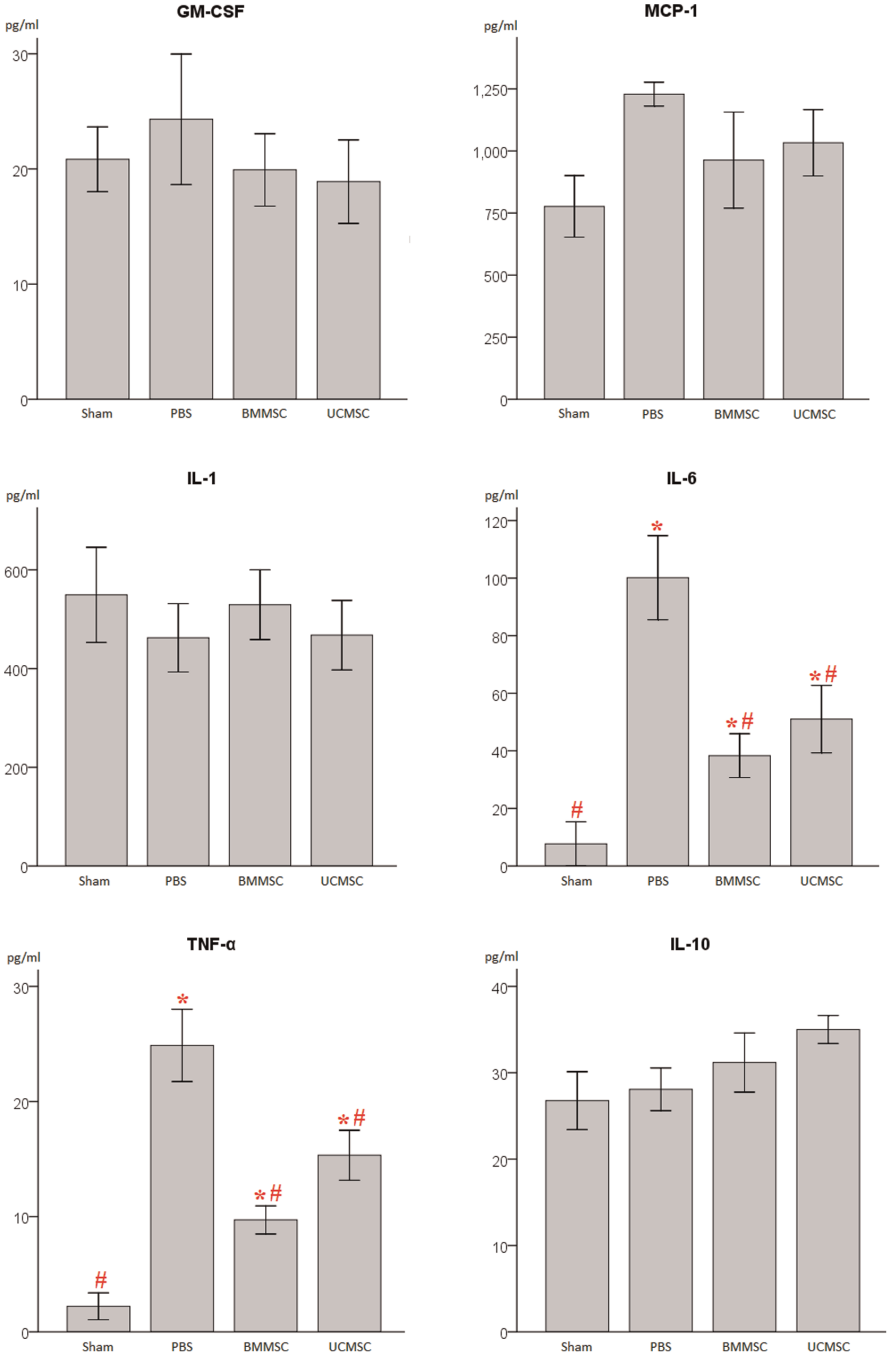
Changes of circulating inflammation-associated cytokine profiles in rats 18 hours after CLP. Compared with sham-operated rats, serum levels of IL-6 and TNF-α were elevated in rats undergoing CLP (i.e. BMMSC, UCMSC and PBS groups). Compared with rats receiving PBS only after CLP, the levels of IL-6 and TNF-α were significantly lower in rats receiving MSCs, either BMMSCs or UCMSCs. There were no significant differences in these two parameters between rats receiving BMMSCs and UCMSCs. There were no significant differences in serum levels of GM-CSF, MCP-1, IL-1 and IL-10 among sham control, BMMSC, UCMSC and PBS groups. Data are presented as mean ± SEM. n = 6–9 rats/group. *p<0.05 versus sham control group. # *p*<0.05 versus PBS group.

To assess the changes of inflammation-associated cytokine profiles after MSC administration during sepsis, serum concentrations of GM-CSF, MCP-1, IL-1, IL-6, TNF-α, and IL-10 were measured 18 hours after CLP or sham operation (Figure 3). Compared with sham-operated rats, serum levels of IL-6 and TNF-α were elevated significantly in rats undergoing CLP, whether receiving MSCs or not. It is worth noting that serum IL-6 and TNF-α levels were significantly lower in rats receiving BMMSCs or UCMSCs than rats receiving PBS only after CLP. These results may implicate that MSCs could rescue rats from the exacerbated inflammatory status after CLP-induced sepsis. There was no significant difference in the serum level of any measured cytokine between rats receiving BMMSCs and UCMSCs after CLP.

### Changes of peripheral blood mononuclear cells in rats with sepsis after MSC administration

**Figure 4:**
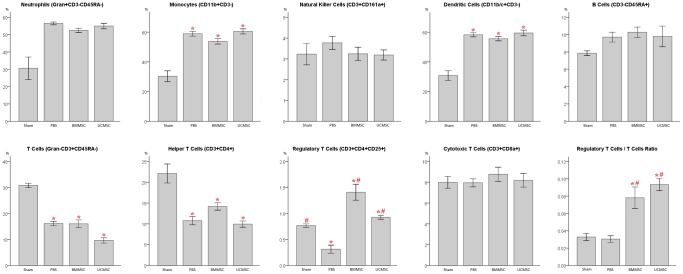
Analysis of peripheral blood mononulcear cells in rats 18 hours after CLP. Compared with sham-operated rats, the percentages of circulating CD11b+CD3- monocytes and CD11b/c+CD3- dendritic cells were elevated in rats undergoing CLP (i.e. BMMSC, UCMSC and PBS groups). Compared with rats receiving PBS only after CLP, the percentage of circulating CD3+CD4+CD25+ Treg cells was significantly higher in rats receiving BMMSCs or UCMSCs after CLP, and so was the ratio of Treg cells/T cells. There were no significant differences in these two parameters between rats of BMMSC and UCMSC groups. Data are presented as mean ± SEM. n = 6–9 rats/group. *p<0.05 versus sham control group. #*p*<0.05 versus PBS group.

To evaluate the activation of the immune system during the inflammatory process induced by CLP, a panel of peripheral blood mononuclear cells, which may participate in the innate or adaptive immune network, were measured 18 hours after CLP or sham operation by their specific surface markers (Figure 4). Compared with sham-operated rats, the percentages of circulating CD11b+CD3- monocytes and CD11b/c+CD3- dendritic cells were elevated significantly in rats undergoing CLP, indicating the activation of the innate immune system after CLP-induced inflammation. Of importance, the percentage of circulating CD3+CD4+CD25+ Treg cells and the ratio of Treg cells/T cells were significantly higher in rats receiving BMMSCs or UCMSCs than rats receiving PBS only after CLP, suggesting that the actions of MSCs may be associated with CD3+CD4+CD25+ Treg cells in this model. There was no significant difference in any peripheral blood mononuclear cells between BMMSC and UCMSC groups.

### Restoration of Treg cell suppressive function in rats with sepsis after MSC administration

**Figure 5:**
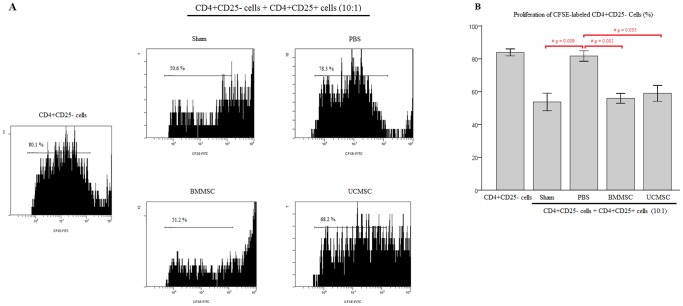
Treg cell suppressive function in rats 18 hours after CLP. (**A**) Analysis of CFSE-labeled CD4+CD25- cell proliferation by flow cytometry after pre-treatment with a IL-2/CD3/CD28 mixture was shown. (**B**) Proliferation of CFSE-labeled CD4+CD25- cells was decreased when cocultured with CD4+CD25+ Treg cells from rats of Sham, BMMSC, and UCMSC groups. The suppressive capacity of CD4+CD25+ cells from rats receiving PBS only after CLP nearly disappeared. Data are presented as mean ± SEM. n = 6–9 rats/group. #*p*<0.05 versus PBS group.

In addition to an increase in the percentage of circulating CD3+CD4+CD25+ Treg cells in the peripheral blood, we further demonstrated the restoration of Treg cell suppressive function in rats with sepsis after MSC administration. As shown in Figure 5, proliferation of CFSE-labeled CD4+CD25- cells was suppressed when cocultured with isolated CD4+CD25+ Treg cells from rats receiving BMMSCs or UCMSCs after CLP. In contrast, the suppressive capacity of CD4+CD25+ Treg cells from rats receiving PBS only after CLP was significantly decreased. These results implicated that immunosuppressive function of Treg cells could be restored by administration of MSCs. Our study provides the first evidence for the increase in Treg cell suppressive function after MSC administration in a septic animal model.

### Detection of the transferred human MSCs in recipient rats

**Figure 6:**
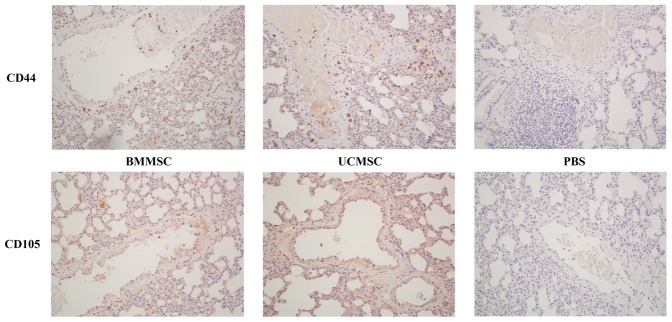
Evaluation of lung immunohistochemistry in rats 18 hours after CLP. Positive cells for CD44 or CD105 which represented the transferred human MSCs can be found in the perivascular interstitial area 14 hours after administration of BMMSCs or UCMSCs (200×).

Further, we tried to find where the transferred human MSCs homed to. At 14 hours after administering BMMSCs or UCMSCs to rats, no cells with CD44 or CD105 expression which represented the transferred human MSCs can be detected by flow cytometry in the peripheral blood. While positive cells for CD44 or CD105 were found in the perivascular interstitial area of the lung sections from rats 14 hours after MSC administration (Figure 6).

## Discussion and Conclusion

Dysregulation of a variety of immune cells in response to sepsis, which is associated with increased rates of morbidity and mortality in septic patients, has been demonstrated [20], [21]. MSCs have been found to modulate immune functions [3]–[8]. In our previous study, we found that UCMSCs could effectively treat severe graft versus host disease after hematopoietic stem cell transplantation, which is a paradigm of immune-mediated host tissue damage [16]. Administering MSCs to mice after CLP was reported to improve survival and organ function [9]–[12], implicating that MSCs may bring immune responses back into balance and attenuate self-tissue damage during sepsis. Regarding how MSCs exert their immunoregulation during sepsis, most investigations focused on the effects of MSCs on neutrophil or monocyte/macrophage function [9]–[12]. Compared with rats receiving PBS only after CLP, the present study showed that the percentage of circulating Treg cells and the ratio of Treg cells/T cells were elevated significantly in rats receiving MSCs after CLP. Further study regarding Treg cell function demonstrated that the immunosuppressive capacity of Treg cells from rats with CLP-induced sepsis was diminished, but could be restored by administration of MSCs. Consistent with the previous reports that Treg cells can control the production of pro-inflammatory cytokines during infection [9], [22]–[24], we found that levels of serum IL-6 and TNF-α decreased in rats receiving MSCs compared with rats receiving PBS only after CLP. This work provides support for the involvement of Treg cells in the immunoregulatory effects of MSCs during sepsis.

Treg cells play an important role in the regulation of immune responses. During infectious processes, Treg cells can suppress the activation of naive autoreactive CD4 helper and CD8 cytotoxic T cells which have the potential to attack the body's health tissues, and control the production of pro-inflammatory cytokines [22], [23], [25]. And thus minimize the collateral tissue damage. In CLP-induced septic mice, adoptive transfer of in vitro-stimulated Treg cells had positive effects on bacterial clearance and survival [26], and the suppressive capacity of Treg cells was prerequisite for the recovery from severe sepsis [27]. The complex interactions between MSCs and Treg cells, two important components of peripheral tolerance in the immune system, have been investigated. MSCs were demonstrated to generate Treg cells and regulate their function [28]–[34], but it has not been reported whether MSCs also can exert their immunomodulatory capacity via Treg cells during sepsis. In the present study, administration of BMMSCs or UCMSCs to septic rats could increase the percentage of circulating Treg cells, the ratio of Treg cells/T cells, and the suppressive function of Treg cells. Thus, levels of serum IL-6 and TNF-α, indices of acute inflammation, decreased. Here, we first reported the alterations in number and function of Treg cells after MSC administration during sepsis in an animal model. And this work provides valuable evidence for the association of Treg cells and MSC-mediated immunomodulation during sepsis.

A broad spectrum of factors produced by MSCs have been reported to affect cell performance, including insulin-like growth factor, hepatocyte growth factor, epidermal growth factor, vascular endothelial growth factor, stromal cell-derived factor-1, IL-10, IL-8, IL-6, prostaglandin E2, etc [35]–[37]. Although cross-species effects have been demonstrated from administration of xenogeneic MSCs, the mechanisms by which MSCs exert their cross-species activity remain unclear. Additionally, there is substantial evidence that infused MSCs have higher engraftment efficiencies within sites of inflammation or injury [38]. In the absence of tissue damage, a large number of systemically administered MSCs was found to lodge in the pulmonary vascular bed [39]. In the present study, the transferred MSCs cannot be detected in the peripheral blood 14 hours after administration but they were found in the pulmonary perivascular interstitium. MSC homing soon to the lung in the recipient rat after systemic administration may be important for amelioration of lung injury. It remains difficult to determine how long the transferred MSCs survive in the recipients. Although no evidence of donor MSCs in the majority of patients 6 months after MSC administration, MSC chimerism still can be demonstrated in several patients at such a long time after administration [40], [41].

For clinical use, a key factor is the origin of MSCs to be expanded in vitro. Bone marrow is considered as the traditional source, but MSCs can also be isolated from other tissues. In our previous studies, we found that UCMSCs can promote hematopoietic engraftment after hematopoietic stem cell transplantation [15], [42] and treat refractory graft-versus-host disease effectively [16]. While the properties of UCMSCs may be similar to their bone marrow counterparts, their characteristics and functional importance in sepsis also need to be investigated. In the present study, BMMSCs and UCMSCs showed a similar in vitro-cultured morphology and surface marker expression. It is of interest that UCMSCs exhibited stronger potential for osteogenesis but lower for adipogenesis than BMMSCs. In CLP-induced septic rats, UCMSCs appeared to have similar effects as BMMSCs. As to be easily harvested and efficiently cultured, umbilical cord might represent a feasible source of MSCs for clinical application.

The ability of MSCs to fine-tune immune responses has led to the idea that MSCs could be attractive candidates of cell-based therapy for infection control and immune regulation. Much still remains to be discovered and further works are warranted.
